# Considering Performance in the Automated and Manual Coding of Sociolinguistic Variables: Lessons From Variable (ING)

**DOI:** 10.3389/frai.2021.648543

**Published:** 2021-04-29

**Authors:** Tyler Kendall, Charlotte Vaughn, Charlie Farrington, Kaylynn Gunter, Jaidan McLean, Chloe Tacata, Shelby Arnson

**Affiliations:** ^1^Linguistics Department, University of Oregon, Eugene, OR, United States; ^2^Language Science Center, University of Maryland, College Park, MD, United States; ^3^English Department, North Carolina State University, Raleigh, NC, United States

**Keywords:** English variable (ING), impressionistic coding, automated coding, classification, machine learning, forced alignment, sociolinguistic variables

## Abstract

Impressionistic coding of sociolinguistic variables like English (ING), the alternation between pronunciations like *talkin'* and *talking*, has been a central part of the analytic workflow in studies of language variation and change for over a half-century. Techniques for automating the measurement and coding for a wide range of sociolinguistic data have been on the rise over recent decades but procedures for coding some features, especially those without clearly defined acoustic correlates like (ING), have lagged behind others, such as vowels and sibilants. This paper explores computational methods for automatically coding variable (ING) in speech recordings, examining the use of automatic speech recognition procedures related to forced alignment (using the Montreal Forced Aligner) as well as supervised machine learning algorithms (linear and radial support vector machines, and random forests). Considering the automated coding of pronunciation variables like (ING) raises broader questions for sociolinguistic methods, such as how much different human analysts agree in their impressionistic codes for such variables and what data might act as the “gold standard” for training and testing of automated procedures. This paper explores several of these considerations in automated, and manual, coding of sociolinguistic variables and provides baseline performance data for automated and manual coding methods. We consider multiple ways of assessing algorithms' performance, including agreement with human coders, as well as the impact on the outcome of an analysis of (ING) that includes linguistic and social factors. Our results show promise for automated coding methods but also highlight that variability in results should be expected even with careful human coded data. All data for our study come from the public Corpus of Regional African American Language and code and derivative datasets (including our hand-coded data) are available with the paper.

## Introduction

Since the earliest days of variationist sociolinguistic research (e.g., Labov, 1963, 1966; Wolfram, 1969; Trudgill, 1974), variable pronunciations in speech collected from communities of speakers have been the basis for much research into the principles and processes of language variation and change. A key methodology in this tradition involves the impressionistic coding of sociolinguistic variables (Wolfram, 1993) – such as determining whether a post-vocalic /r/ was vocalized or rhotic (e.g., *guard* as [ga:d] vs. [gard]) or a word final -*ing* in words like *talking* was produced as -*in* or -*ing* – and making quantitative comparisons within and across speakers in the use of these variables. This work has led to key observations about the *orderly heterogeneity* of language (Weinreich et al., 1968), the systematicity underlying the social and linguistic bases for language variation and change. One bottleneck in sociolinguistic research, especially as opportunities increase to study larger and larger collections of spoken language, has been the immense work that goes into coding sociolinguistic variables. While research on some variable phenomena, like vowels and sibilants (e.g., Labov et al., 1972; Stuart-Smith, 2007; see Kendall and Fridland, 2021), has been advanced by acoustic phonetic analysis, many pronunciation features of interest to sociolinguists, like coronal stop deletion (e.g., Guy, 1980; Hazen, 2011), variable rhoticity or *r-*lessness (e.g., Labov, 1966), final stop devoicing (Farrington, 2018, 2019), and velar nasal fronting or variable (ING) (e.g., Tagliamonte, 2004; Hazen, 2008), the variable under focus in this paper, have continued to rely on careful impressionistic coding by analysts.

Techniques for automating the measurement and coding of sociolinguistic data have been on the rise for the past couple of decades, and parallel developments in automation for other areas of the phonetic sciences (such as the phonetic transcription of large corpora; see e.g., Van Bael et al., 2007). Sociophonetic analyses of vowels, in particular, have seen major methodological advances via popular software like the Forced Alignment and Vowel Extraction suite (FAVE; Rosenfelder et al., 2014, see e.g. Labov et al., 2013 for a large-scale example of its use), and efforts have been ongoing to automate other sociophonetic workflows (Sonderegger et al., in progress). These methods have almost entirely replaced the impressionistic coding for such features, which was a mainstay of early sociolinguistic research (e.g., Labov, 1963, 1966; Trudgill, 1974). The success of these methods for particular features, and the degree of appropriateness of acoustic analysis (as opposed to impressionistic coding) more generally, has hinged on the field's ability to identify acoustic dimensions that relate reliably to the auditory impressions of listeners. Features like vowels and sibilants have relatively straightforward acoustic cues, and for features like these, the field has moved over time to view acoustic measures as more useful than the impressionistic coding of analysts (though we further discuss the implications of such a move, which removes the consideration of auditory importance, in the section Determining the Realization of Pronunciation Variables). This paper focuses on the case of sociolinguistic variables that do not have straightforward acoustic cues, like variable (ING), which have remained the domain of impressionistic, categorical coding.

Corpus phonetic approaches (Liberman, 2019; Kendall and Fridland, 2021: chapter 8) have been growing in popularity and a turn to “bigger data” somewhat necessitates an ability to code data in more cost- and time-efficient ways. Thus, the application of automated approaches to the coding of sociolinguistic variables that typically require manual categorical coding represents an important area for methodological improvement. Some work has engaged in this problem, especially in the phonetic sciences broadly (e.g., Van Bael et al., 2007; Schuppler et al., 2011), although relative to the automatic measurement of features like vowels, as just discussed, efforts for the coding of many sociolinguistic variables are not as advanced. To our knowledge, studies thus far have explored automated techniques for coding the deletion of /n/, /r/, and /t/, as well as schwa deletion and insertion, in Dutch (Kessens et al., 1998; Wester et al., 2001) and, for English, /l/ darkness (Yuan and Liberman, 2009, 2011a), post-vocalic *r-*lessness (McLarty et al., 2019; Villarreal et al., 2020), features of /t,d/ (Bailey, 2016; Villarreal et al., 2020), and, the focus of this paper, variable (ING) (Yuan and Liberman, 2011b), but such work is in its relative infancy and these prior studies, as well as the current paper, set the stage for further advancement.

Two broad approaches have been proposed for the automatic coding of categorical pronunciation features. The first, as proposed and implemented by Kessens et al. (1998), Wester et al. (2001), and Yuan and Liberman (2009, 2011a,b) involves forced alignment systems, which, utilizing automatic speech recognition (ASR) techniques, are typically used to transform an orthographic representation of speech to a time-aligned phone-level representation. While forced alignment was not designed initially with the goal of determining which of different pronunciation variants was produced by a speaker, its underlying algorithms provide key mechanisms for such purposes. The second broad approach is the use of machine learning classification procedures, which are designed to learn patterns in data and associate those patterns with classes of objects or outcomes. In purely computational terms, the coding of many pronunciation variables is a rather straightforward classification task for machine learning. Given some acoustic information along with a set of “gold standard” data for which the correct classification is known, a supervised machine learning algorithm can extract patterns of association in the acoustic data to determine likely groupings that align with the categories. These classifying models can then be applied to new data to make predictions about the category membership of those instances.

Hand-coded data by trained analysts has often been viewed as the “gold standard” on which machine learning methods should be trained and subsequently the standards by which proficiency of different models is determined. Yet, surprisingly, the field knows little about how human analysts compare to one another in the first place. A major issue in the automated coding of sociolinguistic variables is that, given the continuous nature of production and the context-dependent nature of perception, the ground truth of whether a given token was realized as one variant or the other is often not straightforward, even for human analysts. With a few notable exceptions (e.g., Kessens et al., 1998; Hall-Lew and Fix, 2012), very little work has actually empirically tested the extent to which different human analysts agree in their coding. Further, the approaches by Yuan and Liberman (2009) and McLarty et al. (2019) have raised the possibility that the need for human coded data training data can be avoided altogether, by taking advantage of other phonological patterns available in language data from outside the variable context. This raises questions about the necessity of human coded training data vs. ways of harnessing properties of other “variable-adjacent” data for training purposes and the resulting performance of models.

Our paper is motivated by the fact that a wide range of machine learning algorithms are now available that excel at tasks relevant to automatic coding of speech features. Yet, for the successful computational automation of the coding of sociolinguistic variables, several important questions remain outstanding before any widespread adoption can take place. For instance, for any particular situation, what is the most appropriate, or most successful, automated approach of the many available? Further, for supervised approaches, what are the most appropriate training data to lead to successful performance? What hand-labeled data are sufficient as the “gold standard” training data? And, perhaps most importantly, on what basis should the algorithm's performance be assessed? What counts as “successful,” and by what metric? A growing set of techniques have been developed that would seem appropriate for automatically coding variables, and thus far different approaches have been used and with different types of training data, but, rarely has the performance of different approaches been compared to one another for the same dataset. Our paper directly takes up these questions.

We investigate a set of manual and automated sociolinguistic variable coding procedures, considering the performance (inter-analyst agreement for human coders and accuracy and signal detection performance metrics for automated procedures) and outcomes (resultant statistical patterns in variationist analyses) of human coded data and computationally coded data. We implement a series of automated coding procedures following up on techniques and suggestions in recent literature and investigate the influence of different approaches to training data on the outcomes of the procedures.

Our investigation focuses on the English sociolinguistic variable (ING), the alternation of forms like *talking* with *talkin'*. (ING) has been a central variable of interest in sociolinguistics and has fueled a wide range of theoretical and methodological advances over the past half-century. (ING) has remained a feature coded by hand in sociolinguistic research and represents an important test case for automatic variable coding because it does not have well-documented acoustic parameters that correspond with its perceived realization. That said, it is also one of the few sociolinguistic variables that has previously been addressed through automated coding techniques, with Yuan and Liberman's (2011b) study showing promise for the use of forced alignment-based automatic coding methods. For our investigation, we use data from the public Corpus of Regional African American Language (CORAAL; Kendall and Farrington, 2020a). CORAAL provides a large amount of spontaneous speech material for the development and testing of analytic methods and provides data that we can share with this paper. Additional datasets derived from CORAAL (including our hand-coded data) as well as processing scripts are available as Supplementary Material to this paper.

The rest of the paper is organized as follows. In the section Background, we provide further background on (ING) and on the manual and automatic coding of pronunciation features in current sociolinguistic work. We then provide more information about our data in the section CORAAL and its (ING) Data. The section Manual Coding of (ING) in the CORAAL Data describes our hand-coding procedures and the results of inter-analyst agreement assessments, which provide important baseline information for manual coding of sociolinguistic variables generally and the characteristics of our training and test data for assessing automatic coding procedures. The section Coding via Forced Alignment presents a forced alignment-based approach to automatically coding (ING) and its results. The section Coding via Machine Learning presents a series of machine learning approaches to automatic coding for (ING) and their results. Finally, the Discussion and Conclusion offers some concluding observations.

## Background

### English Variable (ING)

The variants of variable (ING) are primarily described in sociolinguistic work in terms of the realization of the final nasal segment, as alveolar [n] or velar [η]. Occasionally work has also considered variation in the vowel realization or other consonantal realizations, such as oral releases [ηg] (see e.g., Kendall and Thomas, 2019), however following the majority of work we treat variable (ING) as falling into two primary pronunciation variants, which we describe as -*ing* and -*in*. While variation in (ING) is realized phonologically and occurs across different morphological forms (i.e., both within individual morphemes (-*ing*) and within larger word forms (e.g., *something, during*), the variable has its roots in the morphology of Old English, arising from competition between the historical present participle morpheme -*ende* and the historical verbal noun form -*ung* (Houston, 1985; Tagliamonte, 2004). Importantly for our present purposes, monosyllabic words (e.g., *thing, king*) are not variable and therefore not considered a part of the variable (ING). A number of papers provide extensive discussions of (ING) and its history; readers are encouraged to refer to Hazen (2008) or Kendall and Thomas (2019) for more general background.

A range of linguistic factors are known to influence (ING) realizations, including the grammatical category of the (ING) word, with verbal words (e.g., *talking, walking*) more likely to occur with -*in* than nouns and adjectives (e.g., *building, amusing*) (Labov, 1989; Tagliamonte, 2004; Hazen, 2008). Phonological context (proceeding and following environment) effects have been found in some studies but not others (Labov, 2001; Kendall and Thomas, 2019). Word frequency (Forrest, 2017), and other word characteristics (e.g., is the word “learned” or “everyday”; Tagliamonte, 2004), have also been found to play a role in (ING) realizations, although relatively few studies have examined such questions in depth. Additionally, lexical stress patterns, coupled with word length, also play a role in patterns for (ING), so two syllable words have been found to be much more likely to be realized with *-in* than longer words (Kendall, 2013).

Social factors are also known to play a role in patterns of (ING) realization. Many studies find greater use of -*in* by male speakers than female speakers (Labov, 1966; Kendall and Thomas, 2019), and social stratification is the norm, with speakers in lower social class groups using much higher rates of -*in* than speakers in high social class groups (Labov, 1966; Trudgill, 1974; Tagliamonte, 2004). Stylistic factors, such as formality and identity construction, are also known to play a role in (ING) realizations (e.g., Trudgill, 1974; Eckert, 2008; Kendall, 2013), although such within-speaker factors are outside the scope of the present investigation. While (ING) variation is ubiquitous across English varieties, speakers of African American Language (AAL), the variety sampled in our data, generally have high rates of -*in* use (Labov, 1966).

### Determining the Realization of Pronunciation Variables

As described in our introduction, the growth of sociophonetics as a research area has represented an embrace of instrumental techniques for the analysis of pronunciation variation, but impressionistic coding by trained analysts remains the norm for certain variables. Sometimes impressionistic coding is done with “acoustic guidance” (e.g., by consulting spectrograms of tokens during coding) but the principal technique ultimately involves a human analyst making a categorical, auditory judgment about the variable, such as whether an instance of (ING) should be coded as -*in* or -*ing*. This manual, impressionistic analysis has remained a robust and valuable approach for analyzing variation and many consistent patterns have been identified through such data. This paper does not argue against such data, though here we make a couple of observations about their limits.

First, manual analysis of sociolinguistic variables is slow and necessarily small-scale. Transitions to bigger data and large-scale analysis in sociolinguistics are hampered by a reliance on hand-coded data. For instance, Wolfram's (1969) study of AAL in Detroit, MI – still representing one of the largest sociolinguistic community studies undertaken – quantitatively analyzed just 60 of the 728 individuals interviewed in the community (Shuy et al., 1968).

Further, despite some detailed investigations into inter-analyst patterns in related areas of phonetic transcription (e.g., Shriberg and Lof, 1991; Cucchiarini, 1993, 1996), there have been limited investigations of inter-analyst agreement in the coding of sociolinguistic variables (as well as in the acoustic measurement of sociophonetic variables; however cf. Duckworth et al., 2011). The limited studies indicate that inter-analyst agreement rates are often not high, sometimes with aspects of analysts' backgrounds playing a role in their impressionistic determinations for a variable, despite their amount of experience or training. For example, Yaeger-Dror et al. (2009) conducted a study of trained analysts' perceptions of post-vocalic /r/ realizations and found that the analysts' own dialect background influenced their judgments. And, Hall-Lew and Fix (2012) found that different professional linguists applied different thresholds for categorizing /l/ vocalization. Further, and not surprisingly, tokens that were acoustically in-between category norms were the most disagreed upon.

It is valuable to recognize that the task of impressionistic, auditory coding is in fact a kind of (often poorly-controlled) perception task, with an N of one or perhaps a few, albeit with participants (coders) that tend to be highly trained rather than naïve to the variable. Thus, we offer that it is not surprising that analysts' codes are affected by factors known to affect linguistic perception more broadly, like perceptual sensitivity, language background, or the token's context and perceiver's expectations, and that analysts' training seeks to (but does not always) eliminate such biases. For example, in studies of (ING), it has been demonstrated that naïve listeners who were asked to classify *-in*/*-ing* variation reported hearing *-in* more often in grammatical contexts where it is probabilistically more expected in English (Vaughn and Kendall, 2018), and that *-in/-ing* categorization is also affected by listeners' language background (Yuan and Liberman, 2011b).

This raises a question that is often glossed over in sociolinguistics. On the one hand, instrumental methods that measure acoustics can be easy to implement, and do not introduce the kind of bias inherent in relying on individual listeners' judgments, *but*, they gloss over the relevance of the acoustic details to listeners' auditory perception, which is arguably an important component of language in use (see Kendall and Vaughn, 2020). On the other hand, hand-coding methods that rely on coders' auditory perception reflect the reality of the perceptual system's biases, but are harder and slower to implement, and also to replicate. Thus, it is more difficult than it seems to develop and validate automated methods of impressionistic coding: What standards do we, as a field, think are important in assessing whether the system has “done a good job”? That they perform consistently (in comparison to what, human coders)? That they perform in a similar way to human coders (have a harder time with the kind of tokens that humans do)? That they would result in similar macro-level patterns across the speakers sampled (that social and linguistic factors would pattern in an expected way)? We consider these and other points in our assessments throughout this paper.

### Automated Approaches to Coding Pronunciation Variables

That manual analysis is limiting for the growth of sociolinguistic studies is not a new observation and, as mentioned earlier, a handful of studies have applied computational techniques to the domains of traditional by-hand analyses. Across approaches, the basic premise is that a computational model of some kind (whether a machine learning classifier or as a part of a larger ASR workflow within a forced alignment system) learns to differentiate categories based on some source materials (*training data*) and then that model can be applied to new instances of the feature of interest (*test data*).

We focus our consideration on the use of automated coding for specifically sociolinguistic purposes, but we note that this domain of work falls within a larger area of research on the automation and validation of phonetic transcription and speech technologies like forced alignment. Much of this work has not been picked up by sociolinguistic researchers. However, it offers much to the advancement of sociolinguistic methods for both manual analyses (e.g., considerations of agreement in phonetic transcription; Shriberg and Lof, 1991; Cucchiarini, 1996) and automated approaches (e.g., considerations of how automated phonetic transcription systems perform in comparison to human analysts; Wester et al., 2001; Kessens et al., 2003; Binnenpoorte, 2006; Van Bael et al., 2007).

Using forced alignment as a tool for coding variables was one of the first applications of computational methods for automated sociolinguistic variable coding. These approaches rely on the forced alignment's system to differentiate categorical phonetic forms from acoustic information available in the signal. Yuan and Liberman, the creators of one of the first widely used forced alignment tools, Penn Phonetics Lab Forced Aligner (P2FA; Yuan and Liberman, 2008), trained their forced alignment algorithm to differentiate light /l/ and dark /l/ realizations in recordings of oral arguments from the Supreme Court of the United States (Yuan and Liberman, 2009, 2011a). In this study they took advantage of English phonological processes, whereby light and dark /l/ realizations are unambiguous in word initial (light /l/) and word final (dark /l/) position. They then trained their system on a phonological mapping of two phones: L1, light /l/ based on word initial position, and L2, dark /l/ based on word final position. After training on canonical dark and light /l/, the model was then applied to ambiguous tokens (word medial /l/) to assign one of the two labels, L1 or L2, to individual tokens. This innovated a creative solution to one of the hardest issues in automated coding, which is establishing the training data, here based on non-variable canonical representations of the phones.

In a second study – the most direct analog to the focus of the present paper – Yuan and Liberman (2011b) used a similar technique to analyze (ING) realizations in two corpora, adding a supervised learning step where their acoustic models were trained on human-labeled forms of -*in* and -*ing* for (ING) words from the Buckeye Corpus (Pitt et al., 2007). They then tested categorization on a new set of unseen data balanced for -*in* and -*ing* forms. Comparing their system's overall agreement against eight native English speakers' and 10 native Mandarin speakers' agreement across 200 tokens, they found that their approach reliably categorized -*in* and -*ing* with agreement rates comparable to agreement between native English-speaking coders (an average of 85% agreement).

Bailey (2016) extended this kind of work, testing the FAVE-Align (Rosenfelder et al., 2014) system on three variables, *t/d*-deletion, *th*-fronting, and *h*-dropping. Diverging from Yuan and Liberman's work, this study did not explicitly train a new acoustic model on the variable pronunciations or speakers, and instead aligned its British English speech with an American English acoustic model (a typical practice in forced alignment), adding alternative pronunciations for the variables to the dictionary for alignment. The system's outcomes agreed rather well with manual variable codes for *h-*dropping (~85%) and *th-*fronting (~81%) and less well for *t/d-*deletion (~71%) especially in cases of *t/d* presence where inter-analyst agreement was also lower. Despite the less customized training and testing, Bailey's work again demonstrates that forced alignment categorization has overall high levels of agreement with human analysts across variables. However, Bailey also observed that FAVE-Align was sensitive to factors that human analysts were not, with FAVE accuracy decreasing as speech rate increased, while human analysts remained unaffected (though this may be the result of an acoustic model trained on a different variety).

McLarty et al. (2019) used similar reasoning to Yuan and Liberman (2009) to consider whether post-vocalic /r/ realizations could be automatically coded from a model trained on canonical, i.e., non-variable, “adjacent” contexts. Their study used CORAAL, the same public dataset as used in the present study, adopting a more standard approach to supervised machine learning, the use of support vector machine (SVM) models. In this study, McLarty et al. (2019) extracted mel-frequency cepstral coefficients (MFCCs, more on these below) at three time points across three phonological categories: vowels, pre-vocalic /r/ (which is non-variable but acoustically different from post-vocalic /r/), and post-vocalic /r/. They then trained an SVM on oral vowels and pre-vocalic /r/ tokens, and tested classification on post-vocalic /r/ and unseen vowels. The use of the non-variable phones in training was meant to provide an unambiguous representation of mappings between acoustic information and phone categories. They demonstrate overall that the results from an SVM approach applied to a social analysis of variability in CORAAL largely align with previous studies of *r*-lessness in AAL, suggesting that SVMs and the use of “variable-adjacent” phones for training may be a fruitful method for automated data coding.

Most recently, Villarreal et al. (2020) used random forests to classify post-vocalic /r/ and medial /t/ variables in New Zealand English. Unlike McLarty et al. (2019) this study relied on hand-coded tokens as the training data, with 180 acoustic measures for post-vocalic /r/ tokens and 113 acoustic measures for medial /t/ tokens. In addition to finding a good fit between their models and their training data for /r/ and for binary classification of /t/, they show that the output of their classifier predicted the ratings of trained human listeners for new tokens of post-vocalic /r/, both in terms of gradient judgment and binary classification (absent vs. present). In their paper, Villarreal et al. (2020) presented a critical assessment of McLarty et al.'s (2019) approach to training data, questioning the premise that the study's use of oral vowels and pre-vocalic /r/ tokens provided adequate acoustic information for a post-vocalic /r/ classifier and arguing against the use of such extra-variable forms as training data. While their critique raises valuable points about the need for further testing, their comments appear to miss the possible value of such an approach: Training a classifier on pronunciations outside the variable context has the potential to act as a crucial workaround for the key step in any automatic coding algorithm, which is the need for ample and robust training data. Our takeaway from their critique is that the potential use of different kinds of training data need to be tested, validated and strengthened, and on a per-variable and per-context basis, rather than assuming that one approach is inherently flawed.

Our investigation focuses on several key questions that build on these prior foundations in the automated (and manual) coding of sociolinguistic variables. But, before proceeding, we note that our study does not consider all of the important issues. For instance, we consider an unsupervised approach to coding using a state-of-the-art forced alignment system along with a set of supervised machine learning classifiers. However, we do not set up those approaches to compare them fully in an “apples to apples” way. Rather, we implement each in what we believe are typical use-case ways, embracing the rich acoustic model that the aligner is capable of building for our investigation of forced alignment (in section Coding via Forced Alignment). For our machine learning classifiers (in section Coding via Machine Learning), we focus on a set of simpler, mel-frequency cepstral coefficients (MFCCs) as the acoustic measures, without extensive parameterization or transformation. The use of MFCCs are standard in many areas of speech technology including ASR and are known to provide good representation of the acoustic signal for such purposes (Davis and Mermelstein, 1980; Huang et al., 2001). MFCCs represent extracted values (coefficients) from a mel-frequency cepstrum, which, simply put, is a non-linear spectrum of a spectrum. For variable (ING), a feature without standard acoustic measures, we believe that MFCCs are a useful acoustic representation, but we also acknowledge that further testing – into both other potential acoustic measures and the parameters for the MFCC extraction – would be beneficial. Additionally, while many of the previous studies emphasize the role of gradience in assigning values to sociolinguistic variables through the use of probability estimates of token classification (Yuan and Liberman, 2011b; McLarty et al., 2019; Villarreal et al., 2020), we limit our investigations to binary classification of (ING) to assess the general utility of different automated methods.

## Coraal and Its (ING) Data

The data for this project come from the Washington DC components of the public Corpus of Regional African American Language (CORAAL; https://oraal.uoregon.edu/coraal/; Kendall and Farrington, 2020a). CORAAL is a collection of sociolinguistic interview recordings, along with time-aligned orthographic transcription, from a range of community studies focusing on African American Language (AAL), arranged into several components (subcorpora). Two of the main components are from Washington DC and these are the source of data for the present study. CORAAL:DCA contains sociolinguistic interviews from Fasold's (1972) foundational study of AAL in Washington DC recorded in 1968 (Kendall et al., 2018a). CORAAL:DCB contains sociolinguistic interviews conducted during fieldwork led by Minnie Quartey specifically for CORAAL in 2015–2018 (Kendall et al., 2018b). Both CORAAL components include extensive demographic information about the speakers, including their age, gender, and assignment to one of three socioeconomic classes [SECs: 1 (lowest) to 3 (highest)]. The two components, recorded about 50 years apart from one another, reflect some differences in sociolinguistic interview recordings, in terms of both content and recording technology. They also can be expected to involve recordings with different acoustic properties (the DCA interviews were recorded on reel-to-reel tape and digitized in ~2013; the DCB interviews were recorded digitally using modern solid-state recording hardware; see Kendall and Farrington, 2020b). Our investigation uses both sets of recordings together, and thus provides baseline performance information for the classification of tokens from somewhat heterogenous data. For sake of space, we leave considerations of differences between the two components for future work. It should be noted that our paper does not focus on AAL, but all of the speakers examined identify as Black/African American.

(ING) variation in the CORAAL data was the focus of a (2019) paper by Forrest and Wolfram, who used a set of speakers available in an early version of CORAAL to explore this variable. They focused on speakers in age groups 2–4, with a goal of achieving balance across demographic categories. While our data are independent of the tokens impressionistically examined in that work, their paper provides a preliminary view of the patterns in CORAAL. They identified socioeconomic differences in the rates of (ING) variation in both components of CORAAL, with high rates of -*in* use among the lowest SEC group (above 93% in DCB) and decreasing rates among the higher SEC groups, along with an interaction between gender and SEC for DCA, where males used much higher rates of -*in* than females in the lower SEC groups. Grammatical conditioning has been found for (ING) in several varieties of English (Tagliamonte, 2004; Hazen, 2008), where the *-in* variant is more likely in verbs than in forms like nouns and adjectives. In DCA and DCB, Forrest and Wolfram found only weak grammatical effects, although verbs did exhibit the highest rates of *-in* in both components, aligning with other work on (ING). While our data source is the same, we would not necessarily anticipate identical results to Forrest and Wolfram (2019) for methodological reasons. In our study, we included speakers from a wider range of age groups and also extracted our (ING) tokens to code a random sample from all available (ING) tokens of the speakers selected (e.g., we did not implement type/token limits), rather than the sequential, systematic token inclusion procedures typically used in sociolinguistic analyses.

To examine (ING) in CORAAL, we mined the DCA and DCB components for data. All speaker turns containing non-monosyllabic words with word-final “ing” were extracted from the publicly available R version of the corpus text for DCA and DCB. Interviewers from DCA, who for the most part were not African American, and a few tokens from “miscellaneous” speakers, were removed from the dataset. We also extracted words from a separate, phone-level aligned version of the transcripts, generated using the Montreal Forced Aligner (MFA; McAuliffe et al., 2017); this process is described further in the section Coding via Forced Alignment. We merged these two versions of CORAAL to select tokens of (ING) for analysis. In addition to the variable (ING) words extracted from the corpus, words with word final [ın] and [ıη] that are not in the variable context for (ING) (e.g., *in, thin, Chaplin, vitamin* for [ın] and monosyllabic -*ing* words, like *thing, bring, cling, wing*, for [ıη]) were also extracted for comparison with the variable (ING) cases.

For each variable (ING) word and each non-variable *IN* and *ING* word, 12 MFCCs were extracted from four temporal measurement points in each final vowel+nasal portion of the word, 25, 50, 70, and 90% of the vowel+nasal segments' combined duration, following prior work citing the importance of vowel quality in (ING) classification (Yuan and Liberman, 2011b). These were based on the segment alignments from the MFA forced alignment. The MFCCs were extracted using the tuneR package in R (Ligges et al., 2018). Words with final vowel+nasal segments that were ≤50 milliseconds or for which our MFCC extraction process otherwise failed to obtain MFCCs were dropped from the dataset. This left a total of 8,255 *IN* words and 1,436 *ING* words in the non-variable MFCC data and 12,041 (ING) words in the variable data. Preliminary tests assessed a range of different MFCC extraction parameters and their impacts on the later classification steps of our process but we found little impact of minor changes to the MFCC parameters. We do not focus on testing different MFCC time points or window lengths in this paper but our initial investigations indicated that four temporal measurement points for the extraction of MFCCs performed better than tests with two or three time points, even though fewer time points allowed for the inclusion of shorter vowel+nasal segments (so led to an increase in the total number of tokens that could be considered. Data sources and R code along with more information about our procedures, including the specific settings used for e.g., MFCC extraction, are provided as Supplementary Material).

## Manual Coding of (ING) in the Coraal Data

Before considering the ability of automated, computational approaches to code instances of (ING), it is important to assess the nature of such data from the perspective of human coders. As discussed earlier (in the section Determining the Realization of Pronunciation Variables), very little work in sociolinguistics has published accounts of inter-analyst agreement in the coding of variables (cf. Hall-Lew and Fix, 2012). Understanding the degree to which human coders agree about codes for (ING) is important before we can assess the performance of machine coding of the variable. Further, human annotations for gold standard training and test data are a major component of most machine learning classification approaches, so understanding the properties of the human coded data is important for the other steps of our research project.

For the human coded data, 50 tokens were randomly subsampled per speaker from the larger dataset, for 24 speakers. All of the speakers are African American and were selected to include the major demographic categories included in CORAAL's sampling – speaker gender, age, and socioeconomic status – but with an emphasis on the lower SEC groups. Tables 1A,B display the breakdown of speakers. In addition to the 1,200 tokens sampled from these 24 speakers, 100 tokens were randomly subsampled from the main interviewer in the DCB corpus, an African American female in her 30s. This interviewer is by far the speaker with the most recorded speech in CORAAL and we thought including a sample of (ING) data from her speech would be useful.

**Table 1A:** Speakers included in Dataset B from CORAAL:DCA.

	**Socioeconomic group 1**	**Socioeconomic group 2 and 3**
Age group 1 (<19)	DCA_se1_ag1_f_04 (95.8%) DCA_se1_ag1_m_07 (95.8%)	DCA_se2_ag1_f_02 (69.8%) DCA_se2_ag1_m_05 (37.5%)
Age group 2 (20–29)	-	DCA_se3_ag2_f_02^*^ (6.0%)
Age group 3 (30–50)	DCA_se1_ag3_f_02^*^ (34.7%) DCA_se1_ag3_m_01^*^ (89.1%)	DCA_se2_ag3_m_01^*^ (87.8%)
Age group 4 (>51)	-	-

*In parentheses is the percentage use of -in by the speaker based on their 50 tokens in Dataset B*.

* *Also included in Forrest and Wolfram (2019) analysis*.

**Table 1B:** Speakers included in Dataset A (in *gray italic* font) and Dataset B (plain font) from CORAAL:DCB.

	**Socioeconomic group 1**	**Socioeconomic group 2**
Age group 1 (< 19)	DCB_se1_ag1_f_03 (77.1%) DCB_se1_ag1_m_02 (89.1%)	DCB_se2_ag1_f_01 (83.3%) DCB_se2_ag1_m_01 (83.7%)
Age group 2 (20 to 29)	DCB_se1_ag2_f_02^*^ (84.6%) DCB_se1_ag2_m_01^*^ (100%)	DCB_se2_ag2_f_02^*^ (10.0%) DCB_se2_ag2_m_01^*^ (87.2%)
Age group 3 (30 to 50)	DCB_se1_ag3_f_03 (93.9%) DCB_se1_ag3_m_02^*^ (88.0%)	DCB_se2_ag3_f_02 (62.0%) DCB_se2_ag3_m_02^*^ (60.4%)
Age group 4 (>51)	DCB_se1_ag4_f_01 (97.9%) DCB_se1_ag4_m_01 (84.0%)	DCB_se2_ag4_f_05 (83.3%) DCB_se2_ag4_m_01 (94.7%)

*Plus DCB_int_01 (female interviewer, mid 30s; -in: 58.7%)*.

*In parentheses is the percentage use of -in by the speaker based on their 50 tokens in Dataset A or B*.

* *Also included in Forrest and Wolfram (2019) analysis*.

For two of the speakers, DCB_se1_ag2_f_02 and DCB_se1_ag2_m_01 (both in the lowest socioeconomic group and in the 20–29 age group), all seven authors coded each of the tokens. We hereafter refer to this as Dataset A, and we use it to assess inter-analyst agreement patterns for a(n albeit small) dataset coded by more than just a few analysts. For the other 22 speakers and the interviewer, three analysts coded each token. We hereafter refer to this as Dataset B. Thus, for Dataset A we have seven independent ratings for 100 of the (ING) cases and, for Dataset B, three independent ratings for the other 1,200 tokens.

In addition to the hand-coded tokens just described, an additional set of 900 tokens, hereafter Dataset C, were randomly selected from CORAAL:DCA and CORAAL:DCB with no sampling criteria other than that these tokens did not come from interviewers in DCA (who, again, were generally not speakers of AAL) and that did not overlap with the 1,300 tokens sampled for the Datasets A and B. Dataset C includes tokens from 113 speakers, with an average of 8.0 tokens per speaker and a standard deviation of 8.4 (a maximum of 69 for the main DCB interviewer, who is the person with the most speech in the corpus, to a minimum of 1 token each for 11 speakers, who generally are speakers who contribute only small amounts of speech to the corpus). This final set of tokens was coded by two of the authors and is used in some of our analyses as an additional test dataset. We note that as a random sample of the entirety of CORAAL:DCA and DCB, Dataset C is useful for examining the overall patterns that might occur across the complete dataset. It also allows us to test samples of speech from speakers who are not present in any of the data we use for training models. We also note, however, that Dataset C is somewhat artificial as an example of a sociolinguistic dataset, since most sociolinguistic studies will sample speakers in more systematic ways and will not, for example, develop a dataset with such imbalanced tokens across speakers. Nonetheless, we believe that Dataset C provides us additional value as a test case for our automated techniques.

In order to code the tokens, the human analysts worked from spreadsheets of excerpts from orthographic transcriptions, with each excerpt line containing one specified (ING) word. Each line contained a direct link to the audio for the token's utterance via the online interface to the corpus (http://lingtools.uoregon.edu/coraal/explorer/browse.php). Analysts were instructed to listen to the token in context, and code the (ING) tokens auditorily according to the following categories: “G” if the form was clearly *-ing*, “G?” for cases where the analyst believed it was -*ing* but wanted to register a lack of confidence, “N” if the form was clearly *-in*, and “N?” for *-in* but without confidence. Finally, analysts were instructed to use “DC,” for *don't count*, if for some reason the token did not appear to be a good candidate for analysis. There are several reasons a token could be a *don't count* form, ranging from instances where our initial extraction selected tokens that simply were not good for analysis (e.g., the speech overlapped with other simultaneous speech in the recording or the token involved some disfluency on the part of the speaker) to cases where the form was determined to be too unclear to code. Since the (ING) tokens were selected from the corpus by script, the coders were instructed to use DC codes as liberally as necessary and we might expect a higher number of DC cases here than in typical variationist analyses which pre-select tokens for inclusion using more deliberate processes. Aside from these reasons for marking a token as DC, all non-monosyllabic *ing*-final words were included as candidates for the (ING) variable. We note that researchers examining (ING) have implemented different practices regarding some aspects of the variable, such as whether lexical exclusions apply (e.g., excluding words like *anything* and *everything* which tend to favor -*ing* or words like *fucking* which tend to favor -*in*). Our practices follow Hazen (2008) and Kendall and Thomas (2019) in not applying any such exclusions (see also sections English Variable (ING) and Automated Approaches to Coding Pronunciation Variables).

Importantly, we note that all of the authors are trained linguists with varying degrees of research experience with AAL, however none are speakers of AAL. Research experience of the authors ranges from extensive transcription of interviews in CORAAL to research and publications on AAL more broadly. This fact may be one potential factor affecting our coding, as language backgrounds have been observed to influence perceptual categorization of variants. We note, however, that this fact – non-AAL speakers coding AAL data – is not unusual in sociolinguistic studies, so may be representative of a more widespread limitation of impressionistic coding in sociolinguistics. The question of language variety background and inter-analyst agreement in sociolinguistics is outside the scope of our paper, but warrants further attention.

### Dataset A: Inter-analyst Agreement Among Seven Human Coders

We begin by considering the patterns of agreement in Dataset A, the 100 tokens coded by all seven analysts. This is admittedly a small dataset but little sociolinguistic work (or other linguistic annotation description) has reported coding outcomes by more than a few analysts, so we begin by assessing what kinds of agreement coding might yield across all seven analysts.

Of the 100 tokens coded by all of the analysts, 20 tokens received at least 1 DC (*don't count*) code and 9 of the tokens (5 of which overlapped with tokens that also received DC codes) received at least one low confidence (N? or G?) code. In order to simplify the treatment here (i.e., for sake of space), we collapse over the low confidence codes (so N and N? are collapsed to -*in* here and G and G? are collapsed to -*ing*). The breakdown of these codes for the 100 tokens are displayed in Table 2. The high number of forms coded as *don't count* (20% of the data received at least one such vote) is likely a function of the instructions to use DC liberally in order to catch erroneous tokens that were selected by our automated selection procedure (e.g., cases of speaker overlap). Six tokens received 3 or 4 DC votes, which likely indicate that those tokens should indeed be discounted from an analysis, but 10 tokens received only 1 DC vote, which suggests that our DC criteria could have been clearer to the coders. One take-away from the DC forms alone is that subjective decisions about coding involve not only coders' impressions of what form they perceive but also what constitutes a “countable” instance of the variable in the first place.

**Table 2:** (ING) codes for the 100 tokens in Dataset A coded by seven analysts (=how many analysts coded the tokens using a particular code?).

	**0 analysts**	**1**	**2**	**3**	**4**	**5**	**6**	**7 analysts**
-*in*	3	1	2	3	4	8	21	58
*-ing*	76	13	5	0	1	1	1	3
DC	80	10	4	3	3	0	0	0

Fifty-eight tokens were coded as -*in* by all seven analysts. An additional 21 tokens were coded by six of the seven analysts as -*in* (with 12 coded with one -*ing* and the other 9 coded by one analyst as DC). Only three tokens were coded by all analysts as -*ing*, which we take as evidence of the low rate of use of -*ing* by these two working class speakers rather than as something inherent about coding -*ing* cases as opposed to -*in* cases. Most of the other possible outcomes occurred in this small amount of data, with, for instance, one token being coded by four analysts as -*ing* and three analysts as -*in*. Overall, a measure of inter-analyst agreement using Fleiss' Kappa for multiple raters (Conger, 1980) yields a *k* = 0.39 with significantly better agreement than chance for each of the three categories (-*in*: *k* = 0.38, -*ing*: *k* = 0.52; DC: *k* = 0.22). However, the agreement values still fall only in the “fair” to “moderate” agreement range according to many assessments of inter-analyst agreement (Landis and Koch, 1977). Removing the DC cases, a clear source of disagreement among the analysts for the tokens in Dataset A, improves the agreement rates substantially to *k* = 0.54. This small sample coded by many analysts demonstrates that we need to expect some amount of disagreement as normal in manually coded pronunciation variables like (ING).

As reported in Table 1 earlier, the two speakers included in Dataset A were heavy users of -*in*. Removing all tokens which received any DC votes and taking a majority-rules view of the realization – where we take the majority of analysts' codes as the category for a token – only six tokens would be assigned as -*ing* across the two speakers and all were produced by the female speaker, DCB_se1_ag2_f_02 (-*in* rate = 84.6%). The male speaker, DCB_se1_ag2_m_01, had categorical use of -*in*. In retrospect, it would have been more useful to include speakers who were more variable in Dataset A, but we did not, of course, know their rates of use before selecting the speakers for inclusion.

### Dataset B: Inter-analyst Agreement Among Three Human Coders

For further consideration we move to assess the codes generated by three analysts for the other 22 speakers and the interviewer. To do this, we first removed all tokens that were coded as DC by any of the analysts. This removed 65 tokens from the 1,200 tokens coded by three analysts, leaving 1,135 tokens. The breakdown of codes is presented in Table 3. An assessment of the inter-analyst agreement using Fleiss' Kappa yields *k* = 0.77 for the data including the low confidence ratings (N, N?, G?, and G) and *k* = 0.79 if the confidence codes are collapsed (i.e. just assessing -*in* vs. -*ing*). These are high levels of agreement, in the “substantial agreement” range by common rules of thumb. In simpler terms, and collapsing the confidence marks, the coders agree (all three assign the same major code) for 980 tokens (86.3% of the 1,135 tokens).

**Table 3:** (ING) codes for 1,135 tokens in Dataset B coded by three analysts (not including tokens with DC codes).

**Codes:**	**N-N-N**	**N-N-N?**	**N-N?-N?**	**G?-N-N**	**G-N-N**	**G-N-N?**	**G-G?-N**	**G-G-N**	**G-G-G?**	**G-G-G**
N:	697	3	1	2	107	2	5	39	8	271
**%:**	61.4%	0.3%	0.1%	0.2%	9.4%	0.2%	0.4%	3.4%	0.7%	23.9%
	Agree *-in*: 701 (61.8%)			Disagree: 155 (13.7%)					Agree -*ing*: 279 (24.6%)	

Taking a majority-rules view of the coded data – i.e., any tokens with two or more G or G? codes count as -*ing* and two or more N or N? codes count as -*in* – suggests that, overall, the speakers produced 812 (71.5%) of the tokens as -*in* and 323 (28.5%) as -*ing*. These values provide both a useful benchmark for the potential results of automated approaches to coding CORAAL's (ING) data. They also provide a useful starting place for training data for such a coding system. We use Dataset B extensively for training and testing automatic coding routines in the section Coding via Machine Learning.

### Dataset C: Inter-analyst Agreement Among Two Coders

As an additional dataset for assessing the performance of automated coding methods, two analysts coded (ING) for the additional set of 900 tokens from CORAAL. These two raters obtain 88.4% agreement for this second set, with a Cohen's *k* = 0.73. The breakdown of these tokens is presented in Table 4, showing overall rates of -*in* (63.2%) and -*ing* (25.2%), with 11.6% of the tokens as ambiguous, having been coded as *-in* by one analyst and -*ing* by the other. While these tokens are sampled more randomly than the sample in Dataset B, comprising a wider assortment of speakers across all of the CORAAL:DCA and CORAAL:DCB, these rates are taken as comparable to the 71.5% -*in*/28.5% -*ing* rates in Dataset B. Dataset C is used as test data in our assessments of automated coding routines in the section Coding via Machine Learning.

**Table 4:** (ING) codes for 900 tokens in Dataset C coded by two analysts.

**Codes:**	**N-N (Agree *-in*)**	**N-G (Disagree)**	**G-G (Agree -*ing*)**
N:	569	104	227
**%:**	63.2%	11.6%	25.2%

## Coding via Forced Alignment

As a first step toward automatically coding variable (ING) in CORAAL, we submitted CORAAL:DCA and DCB (v. 2018.10.08) to forced alignment, using the Montreal Forced Aligner (MFA; version 1.0). This alignment was done using MFA's train and align option, which creates an acoustic model based entirely on the dataset itself. For the pronunciation dictionary, we provided the Montreal Forced Aligner (MFA) with an edited version of the Carnegie Mellon University pronunciation dictionary that, crucially, included two pronunciation options for each (ING) word (e.g., *bringing* was represented in the pronunciation dictionary supplied to MFA with both B R IH1 NG IH0 N and B R IH1 NG IH0 NG as potential pronunciations). These entries were added to the dictionary using a script, which is included in the Supplemental Material. Speaker adapted triphone training was used in the train and align option in MFA, where speaker differences and context on either side of the phone are taken into account for acoustic models.

Before proceeding, we note that the use of a large, high variability training data set (number of speakers, acoustic quality, etc.) is expected to provide a more robust acoustic model for alignment (McAuliffe et al., 2017). That is, MFA was trained on all of the acoustic information available in DCA and DCB and allowed to assign phone labels to all (ING) words, with no data held out for separate testing. This differs from the training and testing approaches we take up in the section Coding via Machine Learning, but follows typical practice for use of modern aligners like MFA (However, unlike many uses of aligners, and e.g., the approach used by Bailey (2016) to code variables, our MFA acoustic models were trained specifically on CORAAL data and in a way that allowed the model to learn different pronunciations for (ING)). We do this to emulate the standard workflow that we would expect of sociolinguistic studies using forced alignment techniques; since there is no need for hand-coding training data in this unsupervised method, there is not the same motivation for testing the forced alignment system on a held out subset of the data as is the case when using hand-coded training data in our supervised classification techniques. Therefore, we emphasize that we expect MFA to do quite well, since the test dataset is subsumed by the training dataset.

As a first assessment of the codes obtained from the MFA alignment, we consider its performance compared to the human analysts' judgments for Dataset B, with the codes from the three analysts collapsed over confidence ratings (i.e., G and G? → G, N and N? → N). This is shown in Table 5.

**Table 5:** Human codes for Dataset B along with MFA's pronunciation assessment.

**Human codes:**	**N-N-N**	**G-N-N**	**G-G-N**	**G-G-G**	***Totals***
MFA = -*in*	633 (90.3%)	94 (84.7%)	27 (61.4%)	69 (24.7%)	*823 (72.5%)*
MFA = -*ing*	68 (9.7%)	17 (15.3%)	17 (38.6%)	210 (75.3%)	*312 (27.5%)*
*Totals*	*701*	*111*	*44*	*279*	

*Shading indicates cells where human codes and MFA agree*.

Overall, the MFA outputs yield some similarity to the human coders but in some key places differ substantially. In terms of disagreement, MFA indicated a pronunciation of -*in* for 69 (24.7%) of the cases all three human analysts agreed were *-ing* and -*ing* for 68 (9.7%) cases where all three humans coded -*in*. This diverges from the humans for 12.1% (137/1,135) of the tokens. This difference is on par with the disagreements identified among the human coders for both Datasets B (13.7%) and C (11.6%). Further, the overall rates of MFA's assessment of the pronunciations of (ING) are quite similar to those of the human coders, with MFA assigning 72.5% of the (ING) cases as *-in* to the human coders 71.5%.

An additional way to assess the relative output of the forced alignment's phone labels in comparison to human coders is to ask how the outcome of a variationist-style statistical analysis might compare between the two approaches. While the evidence indicates that about 12% of the individual tokens mismatched between MFA and human coders, are the overall patterns similar, especially for factors that sociolinguists tend to be interested in? We focus here on the social determinants of (ING), each speaker's gender, age, and SEC, along with two linguistic factors, the grammatical category of the (ING) word and the length of the word in syllables. For grammatical category, we limit our focus to a binary comparison which we refer to as verb-like (V-like) vs. noun-like (N-like) forms. These were generated based on a part-of-speech tagged version of the CORAAL being developed (Arnson et al. in progress). V-like includes all of the verbal POS tags along with the pronouns *something* and *nothing* and the words (*mother*)*fucking*, which tend to pattern like verbs in having higher rates of -*in*. N-like includes nouns and adjectives along with prepositions (e.g., *during*) and the pronouns *everything* and *anything*, which are known to have lower rates of -*in*. Word length (in syllables) was generated for each word using a script available from Kendall (2013).

Table 6 displays the results for logistic regression models of the (ING) patterns in Dataset B. Model I assesses the majority-rules view of the human coded data, where each (ING) is assigned -*in* or *-ing* based on two or more coders' agreement, with the dependent variable as -*in*. Model II assesses the MFA output for the same tokens, again with -*in* as the dependent variable. The models include random intercepts for speaker and word and test main effects (no random slopes or interactions were tested) for the three social factors and two linguistic factors just mentioned. Word length is included as a continuous predictor; the other factors are categorical and included using simple (dummy coded) contrasts. For socioeconomic status, the reference level is set to SE1, the lowest SEC group. For age group the reference level is set to the oldest speakers, age group 4 (speakers who are 51+ years old). We note that age is modeled as a categorical predictor, using the age group categories provided in CORAAL. (ING) is typically found to be a stable variable in sociolinguistic community studies, not undergoing change. However, (ING) is often found to show age-grading, with middle-aged speakers showing less use of -*in* in comparison to young and old speakers (due in part to linguistic marketplace factors) (see e.g., Wagner, 2012). While a full analysis of (ING) in CORAAL is beyond the scope of this paper, the expectation of such age-graded patterns motivates our inclusion of age as a factor and the inclusion of age through CORAAL's categorical age groups provides a simple means to uncover non-linear age differences in the data that might be missed through a simple linear treatment of age as a continuous predictor.

**Table 6:** Logistic mixed-effect regression models for (ING) in Dataset B (1,135 tokens).

	**Model I: human coders (*****N*** **=** **1,135 tokens)**			**Model II: forced alignment output (*****N*** **=** **1,135 tokens)**		
	**Est**.	**Std. Err**.	***p***	**Est**.	**Std. Err**.	***p***
(Intercept)	4.78	1.32	0.0003^***^	2.24	0.82	0.0060^**^
Corpus (DCB, vs. DCA)	0.68	0.69	0.3278	−0.44	0.38	0.2416
Gender (male, vs. female)	1.29	0.63	0.0391^*^	0.88	0.34	0.0089^**^
AgeGrp (AG1, vs. AG4)	−1.29	1.01	0.2017	−0.23	0.53	0.6566
AgeGrp (AG2, vs. AG4)	−3.73	1.25	0.0028^**^	−1.80	0.66	0.0060^**^
AgeGrp (AG3, vs. AG4)	−1.88	0.98	0.0553.	−0.73	0.51	0.1526
SEC (SE2 or 3, vs. SE1)	−1.28	0.67	0.0568.	−0.37	0.36	0.3079
GramCat (N-like, vs. V-like)	−1.14	0.37	0.0019^**^	−1.08	0.31	0.0005^***^
Word Len (# Sylls)	−0.68	0.29	0.0189^*^	−0.03	0.25	0.9103

*** *p < 0.001*,

** *p < 0.01*,

* *p < 0.05, .p < 0.1*.

There are some notable differences between the human coded and forced alignment coded data, but also a number of similarities. Models do not identify a significant difference between the two CORAAL components. Both models identify the expected difference between verb-like words and noun-like words, with noun-like words significantly disfavoring -*in*. Both models also indicate that age group 2, speakers between the ages of 20–29, are significantly less likely to produce -*in* than the oldest group of speakers. Neither model finds the other two age groups significantly different from the oldest speakers, although the age group 3 speakers (between 30 and 50) come close to a *p*-value of 0.05 in Model I. Neither model identified significance for SEC differences among the speakers, although the human coded data in Model I approach significance. Both models are also similar in identifying a significantly greater use of -*in* by male speakers. The statistical outcomes suggest that the two approaches to coding were somewhat similar in their sensitivity to social patterns in these data (While we don't focus on the substance of these patterns here, they are roughly in line with sociolinguistic expectations, e.g., with greater use of -*in* by males than females and the appearance of age-grading patterns for (ING)). One striking contrary point, however, is that the word length effect is only significant in the human coded data. The fact that the forced alignment data do not capture this statistically significant pattern in the human coded data may suggest a major difference in how human coders treat, and hear, variable (ING) in comparison to the automated alignment algorithm (see also Yuan and Liberman, 2011b; Bailey, 2016).

## Coding via Machine Learning

We turn now to consider machine learning based approaches more directly, where the coding algorithm can be trained specifically around the features of interest. While a host of potential machine classifiers are available, we focus on the two cases that have seen recent use for sociolinguistic variable coding, support vector machines (SVMs) and random forest (RF) classifiers.

SVMs are a supervised machine learning algorithm that have seen widespread use in classification (Boser et al., 1992), as well as recent work in sociolinguistics (McLarty et al., 2019). The basic mechanism of the SVM approach involves a model identifying a hyperplane in a multidimensional feature space that best separates categories based on those features. One key piece of the SVM architecture is the ability to apply different kernel functions, which allow for different kinds of separating hyperplanes between classification categories. There are other parameters that are customized for SVM algorithms, namely the “cost” of constraints violation parameter *C* and, for radial kernels, *gamma*, which determines how much influence the model places on each training example. There is no single best method for how to parameterize an SVM classifier, with most guidance suggesting an empirical approach, determining the best parameters (so-called “tuning”) based on performance for the data and the problem at hand. We used the e1071 package for R (Meyer et al., 2019) interface to the C++ libsvm implementation (Chang and Lin, 2001) for all SVM models.

RFs are an approach that have seen growing use in sociolinguistics more generally, e.g., for the analysis of sociolinguistic data (Tagliamonte and Baayen, 2012). As mentioned earlier, Villarreal et al. (2020) applied RFs for their automatic coding of sociolinguistic post-vocalic /r/ and medial /t/ data. RFs are a procedure that expand upon classification and regression trees, a common recursive partitioning method, generating many individual trees on a dataset to generate a partitioning solution that is generalizable beyond a specific set of data. RFs have fewer parameters to customize than SVMs, but still benefit from model tuning. A number of random forest implementations are available. We used the randomForest package in R (Liaw and Wiener, 2002).

Altogether, we build, tune, and test three types of machine learning classifiers, an SVM with a linear kernel (hereafter “linear SVM”), an SVM with a radial kernel (“radial SVM”), and a random forest (“RF”). For each, we use two kinds of training data. In the first case, in the section (ING) Classification, Using Human Coded Training and Test Data, we proceed through a somewhat typical supervised machine learning case, where we use subsets of the human coded data in Dataset B to train the classifiers. In the second case, the section (ING) Classification, Using Variable-Adjacent Productions as Training Data, we explore the kind of approach proposed by Yuan and Liberman (2009, 2011a) and McLarty et al. (2019), where “variable-adjacent” phonetic material, from outside the variable context, is used as training data. In both cases, we use human coded data for testing the models.

### (ING) Classification, Using Human Coded Training and Test Data

We start by assessing the success of the three classifiers trained on the hand-coded data. To do this, we use a 10-fold cross validation approach, using the 1,135 tokens in Dataset B, which were manually coded by three analysts, in a series of training and testing assessments.

First, Dataset B was trimmed to exclude tokens that did not occur in our MFCC extracted data (most often because they were too short for our MFCC extraction, although in some cases tokens could not be matched due to multiple potential candidates in the same utterance). This removed a large number of tokens (29.2% of the data), leaving 803 tokens. Of these, 501 (62.4%) were coded as -*in* and 302 (37.6%) were coded as *-ing* based on the majority-rules codes for three raters. For -*in*, 429 (85.6%) of the cases were agreed upon by all three coders, with the remaining 72 cases (14.4%) having agreement by two of the three coders. For -*ing*, 268 (88.7%) of the cases were agreed upon by all three coders with the remaining 34 tokens (11.3%) having agreement by two of the coders.

**Figure 1:**
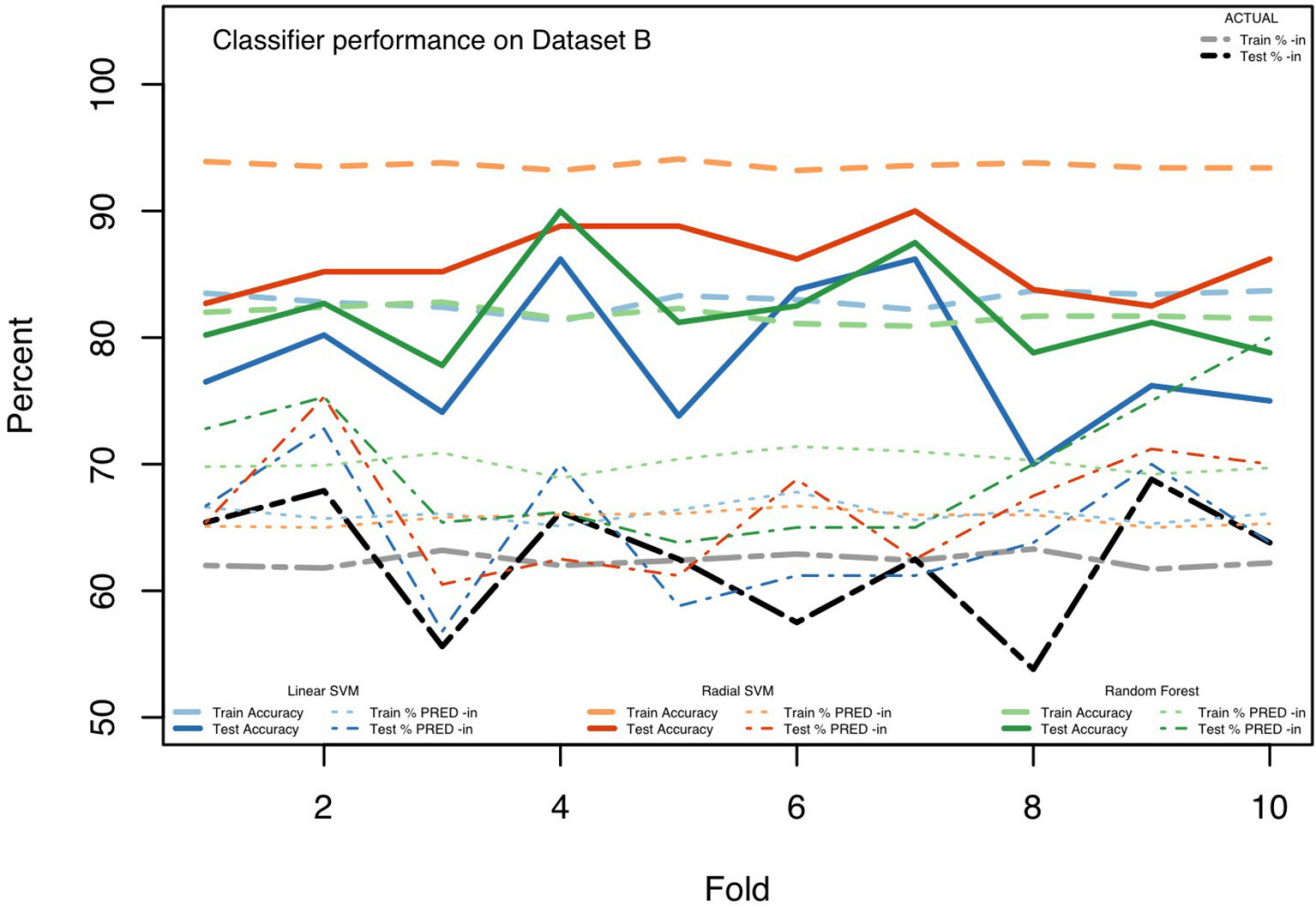
Classifier performance on Dataset B.

Parameters were chosen for the three classifiers by a model tuning step, which conducted a grid-search over candidate settings. After determining parameters based on the entire dataset, the data were randomly divided into 10 “folds,” each containing 10% of the tokens, and then each of the three classifiers was trained, using the 48 MFCCs (12 MFCCs for each of four measurement points), for 9 of these folds (90% of the available hand-coded (ING) tokens), using the majority-rules category (-*in* or -*ing*) as the correct outcome. Each classifier was then tested on the held out 10% of tokens, assessing the model's predictions against the majority-rules coding for those tokens. We repeat this over 10 iterations so that each 10% fold of the data is used as a test case with the other 90% as the training data. Cross-fold validation such as this helps to assess how stable the classifier is to its training and test data. For each iteration, we measure the model's accuracy along with other performance metrics, for both the training data (how well was the model able to fit the training data?) and the testing data (how well was the model able to predict the outcomes for previously unseen data?). We also report the overall percent predicted as -*in* by the models, which helps to show the extent to which each model is over- or under-predicting -*in* vs. -*ing*. Finally, we calculate standard signal detection measures, precision, recall, F1 score, and area under the ROC curve (AUC). These measures provide more insight than accuracy alone, and, are especially valuable since the data are not balanced across -*in* and -*ing* realizations. That is, since 62.4% of the tokens in the (trimmed) Dataset B were coded as -*in* by the human coders, a classifier that always chose -*in* would be accurate 62.4% of the time. Signal detection measures provide more robust performance measures for cases like these. Our reporting of many performance metrics is also meant to provide baseline information for future work on automated coding procedures. Accuracy information from the individual runs of the 10-fold cross validation procedure are displayed in Figure 1 and other performance metrics from across the 10 runs are summarized in Table 7.

**Table 7:** Classification performance for models on Datasets B and C.

	**Dataset B test performance (mean (and std. dev.) across 10 tests; training on 90%, testing on 10%) Actual -*****in*** **rates (mean and std.dev): 62.4% (5.2)**						**Dataset C performance (training on 100% Dataset B, testing on Dataset C)** **Actual -*****in*** **rates: 60.3%**					
	**Pred. -*in* rate**	**Accuracy**	**Precision**	**Recall**	***F*1 score**	**AUC**	**Pred. -*in* rate**	**Accuracy**	**Precision**	**Recall**	***F*1 score**	**AUC**
Linear SVM	64.5% (5.3)	78.2% (5.6)	0.84 (0.05)	0.81 (0.06)	3.25 (0.23)	0.76 (0.06)	70.7%	78.7%	0.91	0.78	3.10	0.75
Radial SVM	66.6% (4.8)	86.1% (2.8)	0.92 (0.04)	0.86 (0.05)	3.46 (0.20)	0.84 (0.04)	69.0%	79.3%	0.90	0.79	3.15	0.76
RF	69.9% (5.6)	82.1% (3.9)	0.92 (0.03)	0.82 (0.06)	3.28 (0.24)	0.79 (0.05)	72.5%	77.7%	0.92	0.76	3.05	0.74

Overall, the classifiers fit the training data with accuracies of 83.0% (linear SVM), 93.6% (radial SVM), and 81.8% (RF) on average. The models' predictive accuracy, their ability to match the human codes for the testing data on each run, is also decent, matching the gold standard codes 78.2% (linear SVM), 86.1% (radial SVM), and 82.1% (RF) on average. We note that these numbers are slightly lower than the agreement between the forced alignment algorithm and the human raters, although the radial SVM's performance is close, only diverging from humans' judgments 13.9% of the time.

We include Figure 1 as an illustration of the performance of the three classifiers, and to underscore the utility of the 10-fold cross validation method. As visible in the figure, across the iterations we see general stability in the models' fits and performance for the training data (the lines for the training data are relatively flat). And the average amount of actual -*in* use is stable across each of the training datasets. This is not surprising given the amount of training data; shifts between which specific 10% folds are excluded do not lead to large changes in the overall proportion of -*in*. Further, the overall good fit, over 93% for the radial SVM, for the training data is somewhat expected. These techniques should be able to model accurately the data provided for training. The more important question is their performance on the testing data.

The testing data, with many fewer tokens (10% of Dataset B, as opposed to the 90% in the training data), are more erratic in the actual rates of -*in* (ranging from 53.8 to 68.8%) across the folds. Predictably, the models appear sensitive to this with, generally, correspondingly variable predictions across the testing folds. The accuracy on test folds varies quite a bit, however, with the worst accuracy at 70.0% for Fold 8 of the linear SVM and the best accuracy, of 90.0%, at Fold 4 for the RF and Folds 5 and 7 for the radial SVM. While the accuracies are at times rather good, the models generally over-predict -*in* in both the training and test data (as seen in the higher lines for the models' -*in* prediction rates in comparison to the actual rates).

As an additional test of the models' performance, we trained each model (a linear SVM, a radial SVM, and an RF) on the entire set of data in Dataset B and then tested these models on the two-analyst Dataset C data. 580 of the 900 tokens of Dataset C were mapped to the extracted MFCC data; the unmapped tokens were removed. 516 of the remaining (ING) tokens had agreement by the two coders (311 -*in*, 205 -*ing*) and we focus on these tokens. The models yield slightly lower performance than they did on Dataset B, over-predicting -*in* at a higher rate than for Dataset B. This difference in performance makes some sense given that Dataset C contains a wider range of speakers, many of whom were not included in the models' training, Dataset B. Nonetheless, the models still obtain accuracies in the high 70% range.

The models all perform relatively similarly, although the radial SVM performs slightly better than the linear SVM and RF models by all measures of performance. The RF model does better than the linear SVM for Dataset B, but not quite as good as the linear SVM for Dataset C. As noted above, all models appear to over-predict -*in* rates, with the RF models doing this the most. Across the board, models' precision is slightly better than their recall.

As a final assessment, we consider the outcomes of logistic regression on the Dataset C tokens. Table 8 provides the output of models similar to (i.e., with the same modeling structures as) those presented in Table 6 but here presenting simple mixed-effect models for the 516 tokens of Dataset C which had MFCC measures and agreement among the two human coders. The only structural difference between the models for Dataset C and B is that for Dataset C the SEC factor was included in the model with three levels (1 lowest (reference level) to 3 highest), as annotated in CORAAL (Dataset C with a wider range of speakers includes a more complete sampling across all three SEC groups, whereas Dataset B only included limited data from SEC 3). Model III presents a model fit to the human coded Dataset C tokens, with the dependent variable being whether the humans coded the token as -*in*. Model IV presents a model fit to the same data with the dependent variable being whether the radial SVM classifier classified the token as -*in*. We present only this classifier's outcomes for space and chose it because it performed slightly better than the other two classifiers.

**Table 8:** Logistic mixed-effect regression models for (ING) in Dataset C (516 tokens).

	**Model III: human coders (*****N*** **=** **516 tokens)**			**Model IV: radial SVM predictions (*****N*** **=** **516 tokens)**		
	**Est**.	**Std. Err**.	***p***	**Est**.	**Std. Err**.	***p***
(Intercept)	5.86	1.40	< 0.0001^***^	4.01	1.00	0.0001^***^
Corpus (DCB, vs. DCA)	0.25	0.61	0.6846	−0.45	0.45	0.3110
Gender (male, vs.female)	1.17	0.55	0.0326^*^	1.90	0.41	0.0000^***^
AgeGrp (AG1, vs. AG4)	−0.10	0.82	0.9024	−2.01	0.64	0.0017^**^
AgeGrp (AG2, vs. AG4)	−1.38	0.86	0.1058	−1.42	0.64	0.0271^*^
AgeGrp (AG3, vs. AG4)	−1.16	0.78	0.1358	−1.25	0.57	0.0283^*^
SEC (SE2, vs. SE1)	−1.09	0.64	0.0888.	−0.36	0.46	0.4380
SEC (SE3, vs. SE1)	−3.67	0.77	< 0.0001^***^	−1.04	0.48	0.0301^*^
GramCat (N-like, vs. V-like)	−0.93	0.49	0.0573.	−0.68	0.38	0.0792.
Word Len (# Sylls)	−1.39	0.42	0.0010^***^	−0.66	0.30	0.0286^*^

*** *p < 0.001*,

** *p < 0.01*,

* *p < 0.05, .p < 0.1*.

Model III, for the human coded data, indicates that gender and socioeconomic status are significant factors in -*in* realization for Dataset C. Males use -*in* at significantly greater rates and the highest SEC group uses -*in* at significantly lower rates than the lowest SEC group, which is the reference level. Model III does not indicate differences among the age groups, but, as Model I did for Dataset B, does show the expected effect for the linguistic factor, word length. The model does not find a statistically significant difference for grammatical category, although the data trend in the expected direction. Model IV, for the radial SVM coded data, also identifies a significant effect for gender, with greater use of -*in* for males. The effects for socioeconomic status are less robust than for the human coded data, but still in the same direction with a significant difference between rates for the highest SEC group in comparison to the lowest. Model IV matches Model III in obtaining a non-significant trend for grammatical category. A major contrast between the two models is that in comparison to the human coded data, the SVM results overemphasize age differences, suggesting significant differences among the age groups that were not seen in Model III. Finally, the SVM-coded data identifies the significant syllable length effect, which the forced alignment model in section Coding via Forced Alignment did not appear to be sensitive to. This is an indication that, unlike the unsupervised forced alignment data, the classifiers trained on hand-coded data did learn associations related to the perceptions of human coders.

### (ING) Classification, Using Variable-Adjacent Productions as Training Data

In this section we return to the idea implemented by Yuan and Liberman (2009, 2011a) and McLarty et al. (2019), where a variable classifier might be trained on related, non-variable but “variable-adjacent” phonetic material. As discussed earlier, this is a novel suggestion with much promise, although, as raised by Villarreal et al. (2020), one that needs extensive validation before we know how much we might trust automated coding procedures that are not trained on data from the same variable contexts that they are used to classify. Here we use the *IN* and *ING* data – from non-variable word final instances of [ın] (words like *begin* and *win*) and [ıη] (monosyllabic words like *thing* and *wing*) – as training data for (ING) classifiers. The key question is whether such forms, which are outside of the variable context and thus should provide stable acoustic evidence for forms phonetically similar to the variable productions of (ING), provide data of value for the training of a variable classifier. If these forms suffice for model training we might be able to get around the costly, slow, work-intensive step of hand-coding training data in the first place. We examine this here, by training a set of classifiers on the non-variable words and then assessing the classifiers' accuracy on the hand-coded (ING) words.

As described in the section CORAAL and its (ING) Data, our extracted MFCC dataset included 8,255 non-variable *IN* words (words like *begin, win*, and the word *in*) and 1,436 *ING* words (e.g., *thing, bring*, and *spring*). There are reasons to expect that these non-variable words will not form perfect approximations of the pronunciation of -*in* and -*ing* variants of (ING), however their basic phonological forms are close to the realizations relevant to variable (ING). Also, and as might be expected, the words in these classes are of greatly varying frequency with e.g., 1,054 tokens of *thing* and 7,860 instances of *in*. This could be a problem for their use as training data. Preliminary testing indicated that using different subsets of the variable-adjacent forms in our training data led to major differences in performance. One area that will need further exploration is how to prune these kinds of datasets for the most appropriate training examples. For the analysis here, we randomly subsampled 1,436 tokens from the *IN* words to match the smaller set of (1,436) *ING* words. This provided a training dataset of 2,872 words, evenly balanced for non-variable *IN* and *ING* words.

We then built three classifiers, again, a linear SVM, a radial SVM, and an RF. Each classifier was trained and tuned using 10-fold cross-validation with training on the categorical *IN* and *ING* data. We then tested each of these trained models against the 803 three-analyst hand-coded (ING) instances in Dataset B and the 516 tokens in Dataset C. The outcomes from these testing runs are presented in Table 9. Performance for these classifiers is slightly lower than the classifiers trained on human coded data (in section Coding via Forced Alignment), especially in their testing performance on Dataset B (comparing left-hand panels of Tables 7, 9). The reduction of performance on Dataset B makes sense given that the earlier models were trained and tested on speech from the same speakers. Testing on Dataset C for the models trained on the non-variable data actually shows much less over-prediction of -*in* and only very small reduction in performance compared to the classifiers trained on Dataset B. In fact, some metrics, such as the F1 Scores for the radial SVM and RF actually slightly improve; in terms of overall predicted rates of -*in*, the models trained on the non-variable tokens get closest to the actual rates for Dataset C. This result likely comes about for a couple of related reasons. First, the non-variable training data included speech from across all of the speakers in the corpus. This likely helped the models in making predictions for Dataset C, which contained variable (ING) tokens from across a wide range of speakers. Dataset B, with tokens selected from only a subset of speakers, was less useful as training data in this way, even though the hand-coded data provided clearer evidence for the models about the mappings between the features (MFCCs) and variants of (ING). Thus, as we will return to, each of the approaches here appears to have advantages, as well as disadvantages.

**Table 9:** Performance of the three classifiers for classification of variable (ING).

	**Dataset B test performance (training on non-variable** ***IN*** **and** ***ING*** **data)** **Actual -*****in*** **rates: 62.4%**						**Dataset C performance (training on non-variable *IN* and *ING* data) Actual -*in* rates: 60.3%**					
	**Pred. -*in* rate**	**Accuracy**	**Precision**	**Recall**	***F*1 score**	**AUC**	**Pred. -*in* rate**	**Accuracy**	**Precision**	**Recall**	***F*1 score**	**AUC**
Linear SVM	64.6%	69.6%	0.77	0.75	2.99	0.67	61.8%	69.8%	0.76	0.74	2.97	0.68
Radial SVM	64.6%	78.8%	0.85	0.82	3.28	0.77	60.3%	75.2%	0.79	0.79	3.18	0.74
RF	70.0%	75.5%	0.86	0.77	3.08	0.72	64.5%	76.4%	0.84	0.78	3.14	0.74

As a final assessment of classifier performance, we once again conduct logistic regression analyses comparing the human analysts' codes to the predictions of the classifier, again focusing on the radial SVM and on Dataset C (We do not include Dataset B simply for space). Table 10 presents the model of this radial SVM output, Model V, along with Model III, of the human coded data (from Table 8). The patterns emerging from the radial SVM's predictions, as indicated by Model V, are similar to those for the human coded data for SEC, gender, and the word length effect (all yielding significant effects), and for grammatical category (not significant). However, like the SVM trained on the hand-coded data, the SVM here also identifies significant age group differences that do not emerge among the human coded data. Most notably, unlike any of the other models, Model V identifies a significant difference between the CORAAL components, with DCB speakers using less -*in* than DCA speakers.

**Table 10:** Logistic mixed-effect regression models for (ING) in Dataset C (516 tokens) (Model III repeated from Table 8).

	**Model III: human coders (*****N*** **=** **516 tokens)**			**Model V: radial SVM predictions (*****N*** **=** **516 tokens)**		
	**Est**.	**Std. Err**.	***p***	**Est**.	**Std. Err**.	***p***
(Intercept)	5.86	1.40	< 0.0001^***^	3.47	0.75	< 0.0001^***^
Corpus (DCB, vs. DCA)	0.25	0.61	0.6846	−0.69	0.32	0.0334^*^
Gender (male, vs.female)	1.17	0.55	0.0326^*^	0.66	0.27	0.0155^*^
AgeGrp (AG1, vs. AG4)	−0.10	0.82	0.9024	−1.11	0.44	0.0126^*^
AgeGrp (AG2, vs. AG4)	−1.38	0.86	0.1058	−1.30	0.43	0.0026^**^
AgeGrp (AG3, vs. AG4)	−1.16	0.78	0.1358	−0.44	0.37	0.2362
SEC (SE2, vs. SE1)	−1.09	0.64	0.0888.	0.01	0.32	0.9704
SEC (SE3, vs. SE1)	−3.67	0.77	< 0.0001^***^	−0.83	0.34	0.0140^*^
GramCat (N-like, vs. V-like)	−0.93	0.49	0.0573.	−0.34	0.31	0.2643
Word Len (# Sylls)	−1.39	0.42	0.0010^***^	−0.75	0.25	0.0022^**^

*** *p < 0.001*,

** *p < 0.01*,

* *p < 0.05, .p < 0.1*.

## Discussion and Conclusion

To conclude, each of these assessments has demonstrated that automatic coding algorithms, through both forced alignment algorithms and machine learning classifiers, can perform close to human coders in their ability to categorize the sociolinguistic variable (ING). Overall, the models tend to over-predict -*in* somewhat, but their performance is promising. In addition to achieving generally reasonable accuracy, precision, and recall, we would argue that the statistical model assessments of the sociolinguistic patterns that would be uncovered through any of these datasets tell roughly similar stories, albeit with small substantive differences (e.g., the word length effect missing from the forced alignment coded data, the SVM trained on variable-adjacent data suggesting a difference between the CORAAL components).

Given the similar performance of the automated coding approaches tested in our study, and more generally the techniques used to implement them, we suggest that the most appropriate approach for automatic coding of variable features will depend on the kind of data used, whether there is any hand coding available, and, crucially, the research questions and design of the study. Using any particular machine learning approach, such as SVMs *or* RFs, should take into account what is gained or lost in that choice. For example, with data that has acoustic measures characterized by collinearity, RFs might be preferred (Villarreal et al., 2020). At the same time, forced alignment-based classification holds great promise for use cases where an entire large corpus can be force aligned through a training and alignment process (as we did for CORAAL). This approach may be less useful or appropriate if less speech is available for training the aligner. (Although here we do note that Bailey's (2016) investigation yielded good results for variables in British English data using an aligner trained on American English.)

For (ING) in particular, in terms of the machine learning approaches, both SVMs and RFs performed relatively similarly, although across the board the radial SVM performed best, followed closely by the RFs. Models generally performed well across all performance measures, although we note that model precision was uniformly better than recall. The consistent better performance of the radial SVMs over linear SVMs indicates that automated coding methods should not be used “off the shelf,” without careful testing and adjustment for the problem at hand. It may be that radial SVMs will consistently outperform linear SVMs on (socio)linguistic data – this would not be surprising – but individual projects should assess that empirically.

Further, our approach to more customized classifiers worked slightly better with hand-coded training data than it did with adjacent, non-variable productions as the training data. Thus, and not surprisingly, it would seem most prudent to use validated carefully hand-coded data for model training when it is available – and we would argue that using such data for testing is crucial – but our results should be taken as additional encouraging evidence, building on Yuan and Liberman (2009, 2011a) and McLarty et al. (2019), that using adjacent, non-variable training data can hold good promise in certain cases. Of course, some variables will be more appropriate to examine through this means than others, where it may prove impossible to identify relevant non-variable analogs. Therefore, we encourage analysts considering automating coding to carefully consider the details of their particular use case when determining which method and which type of training data are most appropriate for their situation.

One major take-away from our study is that rather than interpret automated techniques' performance as measured against some notion of perfect accuracy, we find that human coding for the variable also achieves agreement at rates of only about 88%. We need to ask what it would mean for an automated system to perform better than this. To return to questions this paper began with: on what basis should algorithms' performance be assessed? What counts as successful? And, by what metric? It would seem that accuracy as measured against a set of human coded data (especially by a single coder) is not the right metric (unless one's goal is to replicate exactly the coding practices of an individual analyst). Rather, measures of performance, and success, should recognize that “gold standard” sociolinguistic data are inherently variable, not just in the patterns in the data but also in the practices used for assessment, even in the best of cases. Our comments here parallel conclusions from work on other aspects of linguistic coding and annotation, such as phonetic transcription (e.g., Shriberg and Lof, 1991; Cucchiarini, 1996). Regardless of the specific problem, success should ultimately be measured in terms of the adequacy of the resultant data for the purposes at hand. Similar to Reddy and Stanford's (2015) discussion of desiderata for a fully automated vowel measurement system (see also Kendall and Vaughn, 2020 and Kendall and Fridland, 2021: chapter 8), we argue that automated techniques should not seek simply to replace human analysts but rather that they reflect alternative approaches to coding that have advantages and appropriateness for some applications and disadvantages and inappropriateness for others.

Across automated techniques our results largely triangulate toward a reasonable and not unexpected pattern for (ING) in CORAAL. But of course differences between the individual models' performances and the statistical patterns that emerge caution against taking an uncritical view of, for instance, a *p-*value threshold as the measure of patterns in a dataset. That said, we believe the variability in human coded data offers a similar caution. That is, our statistical analyses of human coded (ING) in CORAAL (in Models I and III) also present somewhat different views of the patterns in CORAAL. It would seem that the story they tell in the aggregate provides a more dependable picture of the patterns for this sociolinguistic variable in these data than any single one of the models. This seems to us a useful observation for the larger sociolinguistic enterprise and not just a point relevant to automatic coding procedures.

As a final note, we observe that major advances in other domains of machine learning and artificial intelligence, such as automatic speech recognition, have come about through the development of larger and larger “gold standard” training datasets. We stress the importance and value of carefully hand-coded datasets, along with the understanding of the variation inherent in auditory coding. Until automated procedures have been extensively validated for a wide range of features and datasets – something that appears to be still rather far off in the future – a bottleneck in the advancing of automated procedures will remain the availability of hand-coded training data. Our study has focused on relatively small training data, and this seems to us an important area for sociolinguistics at present, since large, reliable human coded datasets for variables like (ING) are unlikely to be available in the immediate future. However, it stands to reason that the performance of these kinds of automated classification systems will be improvable with larger training data, which we believe presents a call for greater data-sharing and organized efforts toward open science in the field. Thus, readers will find our datasets, as well as code, included in the Supplementary Material with this paper.

## Data Availability Statement

The datasets presented in this study come from the public Corpus of Regional African American Language, which is available at https://oraal.uoregon.edu/coraal. All derived data along with processing code are provided as Supplementary Material with this paper.

## Author Contributions

TK and CV conceived of and designed the study, with collaboration from CF, KG, JM, CT, and SA. TK, CV, KG, and CF drafted and revised the paper, with help from JM and CT. JM and CT contributed the most human data coding to the project, with TK, CF, CV, KG, and SA also contributed data coding. SA, with help by TK and JM, generated part-of-speech annotations used in the paper. CF managed the forced alignment process. TK conducted the machine learning procedures, with help from CV, KG, and CF. All authors contributed to the article and approved the submitted version.

## Conflict of Interest

The authors declare that the research was conducted in the absence of any commercial or financial relationships that could be construed as a potential conflict of interest.

## Supplementary Material

This includes code and data to replicate and further explore the studies presented here. See the Guide to Supplementary Material for more details.
