# Supplementary material for: Considering Performance in the Automated and Manual Coding of Sociolinguistic Variables: Lessons From Variable (ING)
Source: Front Artif Intell. 2021 Apr 29;4:648543. doi: 10.3389/frai.2021.648543 (PMC8117961; doi:10.3389/frai.2021.648543)
Supplement: Supplementary file 1 [file Data_Sheet_1.PDF]

Considering performance in the automated and manual coding of sociolinguistic variables: Lessons from variable (ING)

Tyler Kendall, Charlotte Vaughn, Charlie Farrington, Kaylynn Gunter, Jaidan McLean, Chloe Tacata, and Shelby Arnson

Guide to Supplementary Materials

As Supplementary Materials, we provide a series of data files and scripts that can be used to replicate, or extend, the processes and analyses from the paper.

CORAAL itself (audio and transcript files in various formants) can be downloaded from its website, https://oraal.uoregon.edu/coraal/. The audio files for CORAAL:DCA and DCB are necessary to reproduce our MFCC extraction. A forced aligned version of the CORAAL transcripts is also available from the CORAAL download site, although we include tab-delimited derivatives of the aligned files here (in CORAAL_Aligned_as_Tables folder), which are sufficient for recreating our processes.

The CORAAL_VARAligned_as_TextGrids folder, included among the Supplementary Materials, houses the output of the Montreal Forced Aligner's (ING) coding for CORAAL:DCA and DCB we generated, in March 2019, for this project. Datasets A, B, C, included here, have the MFA codes included as a column, along with the hand-coded data provided independently by our human coders.

The data files include:

- CORAALDC_IN+ING_Words_wMFCCs_Dec2020.txt
  Tab-delimited text file containing the main MFCC dataset used (includes tokens for which our process did not obtain MFCCs). This file can be reconstructed using the a_generate_INGdata+mfccs_from_aligned_CORAAL.R R script.
- DatasetsA+B_Initial.txt
  Tab-delimited text file containing the hand-coded (ING) tokens for Dataset A and Dataset B, used in paper. The file also contains the (ING) labels from MFA. Dataset B can be merged with the MFCC dataset using the b_setup_handcoded_data_with_mfccs.R R script, to recreate the DatasetB_wMFCCs.txt file.
- DatasetB_wMFCCs.txt
  Tab-delimited text file containing the hand-coded (ING) data in Dataset B after merging with the MFCC data. This file can be recreated using the code in b_setup_handcoded_data_with_mfccs.R.
- DatasetC_Initial.txt
  Tab-delimited text file containing the hand-coded (ING) tokens for Dataset C, used in paper. The file also contains the (ING) labels from MFA. Dataset C can be merged with the MFCC dataset using the b_setup_handcoded_data_with_mfccs.R R script to recreate the DatasetC_wMFCCs.txt file.

- DatasetC_wMFCCs.txt
  Tab-delimited text file containing the hand-coded (ING) data in Dataset C after merging with the MFCC data. This file can be recreated using the code in b_setup_handcoded_data_with_mfccs.R.
- CORAAL_Aligned_as_Tables folder
  This folder contains the output from the Montreal Forced Aligner on CORAAL:DC, after transformation to tab-delimited text files (using the x_batch_convert_to_table.praat script). This is the canonical version of the forced alignment data from CORAAL for DCA and DCB, i.e. without customized training and alignment to identify variant realizations for (ING). The official TextGrids can be downloaded from the CORAAL website, https://oraal.uoregon.edu/coraal/.
- CORAAL_VARAligned_as_TextGrids folder
  This folder contains the output from the Montreal Forced Aligner on CORAAL:DC allowing for variable pronunciations for variable (ING). This was generated in our project by augmenting our basic English pronunciation dictionary (based originally on the CMU Dictionary, but with additional words occurring in CORAAL added) with both *-in* and *-ing* pronunciations for (ING) words. (The R script x_add_INs_to_dict.R will augment a dictionary in this way.) The provided x_batch_convert_to_table.praat script will convert the TextGrids to tab-delimited text files (similar to the contents of the CORAAL_Aligned_as_Tables folder, but with (ING) coded rather than presented as canonical NG). *Nb. Due to the size of this folder, it has been broken into two parts for distribution with the paper. You should download both parts and combine them into one folder.*

The main R script files are:

- a_generate_INGdata+mfccs_from_aligned_CORAAL.R
  This code extracts instances of variable (ING) words along with other word final *-in* and *-ing* words from the tab-delimited text versions of the output of the Montreal Forced Aligner. Using the tuneR package, it extracts MFCCs from the CORAAL audio (which must be downloaded separately, from the CORAAL website) using the settings we used in our study.
- b_setup_handcoded_data_with_mfccs.R
  This code loads, explores, and processes Datasets A, B, and C (the DatasetsA+B_Initial.txt and DatasetC_Initial.txt files), merging Datasets B and C with the MFCC dataset (CORAALDC_IN+ING_Words_wMFCCs_Dec2020.txt). It recreates some of the tables in the paper and includes the analysis of the output of the forced-alignment approach to variable classification on the hand-coded data.
- c_classify_handcoded_datasets.R
  This code builds classifiers on the hand-coded data, using Dataset B for training and Datasets B and C for testing. It recreates some of the tables in the paper and includes the analysis of the output of these classifiers on the hand-coded data.
- d_classify_using_nonvar_training.R
  This code builds classifiers by training on the non-variable data in CORAALDC_IN+ING_Words_wMFCCs_Dec2020.txt. It then tests the models on

Datasets B and C. The code recreates some of the tables in the paper and includes the analysis of the output of these classifiers on the hand-coded data.

We also include two small additional helper scripts:

- x_add_INs_to_dict.R
  This R script will augment an existing pronunciation dictionary with *-in* pronunciations for (ING) words.
- x_batch_convert_to_table.praat
  This Praat script converts from TextGrids to Praat's Table format and saves the Tables as tab-delimited text files in a folder. This script created the files in the CORAAL_Aligned_as_Tables folder. If you run MFA on your own data and want to re-use portions of our code on your own files, or if you want to re-run our code on the (ING) coded MFA output (CORAAL_VARAligned_as_TextGrids), this Praat script should help.
